# Antidepressant Effects of Probucol on Early-Symptomatic YAC128 Transgenic Mice for Huntington's Disease

**DOI:** 10.1155/2018/4056383

**Published:** 2018-08-14

**Authors:** Cristine de Paula Nascimento-Castro, Ana Claudia Wink, Victor Silva da Fônseca, Claudia Daniele Bianco, Elisa C. Winkelmann-Duarte, Marcelo Farina, Ana Lúcia S. Rodrigues, Joana Gil-Mohapel, Andreza Fabro de Bem, Patricia S. Brocardo

**Affiliations:** ^1^Department of Morphological Sciences, Center of Biological Sciences, Universidade Federal de Santa Catarina, 88040-900 Florianópolis, SC, Brazil; ^2^Department of Biochemistry, Center of Biological Sciences, Universidade Federal de Santa Catarina, 88040-900 Florianópolis, SC, Brazil; ^3^Division of Medical Sciences, UBC Island Medical Program, University of Victoria, Victoria, BC, Canada V8W 2Y2; ^4^Department of Physiological Science, Institute of Biological Science, University of Brasilia, Brasília, DF, Brazil

## Abstract

Huntington's disease (HD) is an autosomal dominant neurodegenerative disorder caused by a trinucleotide expansion in the *HD* gene, resulting in an extended polyglutamine tract in the protein huntingtin. HD is traditionally viewed as a movement disorder, but cognitive and neuropsychiatric symptoms also contribute to the clinical presentation. Depression is one of the most common psychiatric disturbances in HD, present even before manifestation of motor symptoms. Diagnosis and treatment of depression in HD-affected individuals are essential aspects of clinical management in this population, especially owing to the high risk of suicide. This study investigated whether chronic administration of the antioxidant probucol improved motor and affective symptoms as well as hippocampal neurogenic function in the YAC128 transgenic mouse model of HD during the early- to mild-symptomatic stages of disease progression. The motor performance and affective symptoms were monitored using well-validated behavioral tests in YAC128 mice and age-matched wild-type littermates at 2, 4, and 6 months of age, after 1, 3, or 5 months of treatment with probucol (30 mg/kg/day via water supplementation, starting on postnatal day 30). Endogenous markers were used to assess the effect of probucol on cell proliferation (Ki-67 and proliferation cell nuclear antigen (PCNA)) and neuronal differentiation (doublecortin (DCX)) in the hippocampal dentate gyrus (DG). Chronic treatment with probucol reduced the occurrence of depressive-like behaviors in early- and mild-symptomatic YAC128 mice. Functional improvements were not accompanied by increased progenitor cell proliferation and neuronal differentiation. Our findings provide evidence that administration of probucol may be of clinical benefit in the management of early- to mild-symptomatic HD.

## 1. Introduction

Huntington's disease (HD) is an autosomal dominant neurodegenerative disorder that affects 10.6–13.7 individuals per 100,000 in Western populations (for review, see [1]). HD results from an expansion of cytosine-adenine-guanine (CAG) trinucleotide repeats in exon 1 of the *HD* gene, leading to an extended polyglutamine tract in the N-terminal of the huntingtin protein [2]. The length of the CAG repeat is inversely correlated with the age of the onset of motor symptoms, which on average occurs in midlife, between 35–50 years of age [3]. The diagnosis of HD is based on the presence of motor symptoms and a positive family history [4, 5]; however, cognitive and behavioral symptoms are common comorbidities in HD [6–8].

Psychiatric manifestations are very common in HD patients, and these include depression, anxiety, and irritability [8]. Sadness and depression appear to be two of the earliest symptoms observed at the onset of the disease, as reported by first-degree relatives [9]. Indeed, major depression is the most common comorbidity in presymptomatic HD carriers [10, 11], while suicide risk is almost four times greater in HD patients than in the general population [12]. Of note, although the depressive phenotype observed in HD patients does not seem to be correlated with cognitive impairment, the development of motor symptoms, or CAG repeat length [13], a depressive phenotype appears to be associated with a more rapid decline in functional ability [14, 15].

Yeast artificial chromosome (YAC) 128 mice express the full-length human *HD* gene with 128 CAG repeats [16] and exhibit reproducible cognitive [17–19] and motor [16, 19, 20] deficits, as well as depressive-like behaviors [20–22] that mimic the disease progression in humans. While the mechanisms underlying the depressive phenotype observed in both HD patients and HD transgenic mice are not fully elucidated, deficits in hippocampal neuroplasticity, namely, hippocampal neurogenesis, are likely to contribute to these mood disturbances in HD. Indeed, a reduction in adult hippocampal neurogenesis has been reported in truncated transgenic HD mice, namely, the R6/1 [23–26], R6/2 [27–31], and N171-82Q [32] lines, as well as full-length transgenic HD YAC128 mice [21, 33]. In addition, treatment with selective serotonin reuptake inhibitors (SSRIs), which have been shown to potentiate neurogenic function in the hippocampus [34–36], has been shown to improve the phenotype and promote neurogenesis in R6/1, R6/2, and N171-82Q HD mice [25, 29, 32], while also attenuating the progression of brain atrophy both in R6/2 and N171-82Q HD mice [29, 32].

Antioxidants are able to positively modulate adult hippocampal neurogenesis [37–39], and recent studies describing the neuroprotective effect of antioxidants on several neurologic disorders have been published [40]. Probucol is a phenolic lipid-lowering compound with antioxidant properties that has been used in clinical treatment and prevention of cardiovascular diseases [41]. However, neuroprotective properties of this compound have been recently described. For instance, probucol has the ability to increase neuroplasticity [42, 43]. Moreover, probucol was shown to promote neuroprotective effects in toxin-induced models of neurodegenerative diseases, including Alzheimer's disease (AD) [42, 44, 45], Parkinson's disease (PD) [46], and HD [47]. In the present study, we investigated the potential beneficial effects of chronic treatment with probucol on the depressive-like behaviors and hippocampal neurogenesis in early- and mild-symptomatic YAC128 HD transgenic mice.

## 2. Material and Methods

### 2.1. Animals

The YAC128 (HD53 line) transgenic mouse colony was maintained on the FVB/N background strain (Charles River, Quebec, Canada). All animals were generated from our local colony with breeding couples generously provided by Dr. Brian Christie (University of Victoria, Canada). Animals were weaned and ear-punched at postnatal day 22 and group-housed according to their sex (maximum of five mice per cage). YAC128 and their WT counterparts were maintained at 20–22°C with free access to water and food, under a 12/12 h light–dark cycle (lights on at 0700 h). All manipulations were carried out between 0900 and 1600 h. For behavioral experiments, animals were placed in the experimental room 24 h before testing to ensure proper acclimatization to the environment. All animal procedures were performed in accordance with the National Institutes of Health Guide for the Care and Use of Laboratory Animals and were approved by the Committee on Ethics of Animal Experimentation of the Federal University of Santa Catarina (Florianópolis, Brazil; protocol number: PP00944). All efforts were made to minimize animal suffering and to reduce the number of animals used in these experiments.

DNA was extracted from mouse ear tissue, and genotyping was performed by polymerase chain reaction (PCR), using primers for detection of YAC LYA (left YAC arm) and RYA (right YAC arm), as recently described by us [21].

### 2.2. Drugs and Treatments

**Figure 1:**
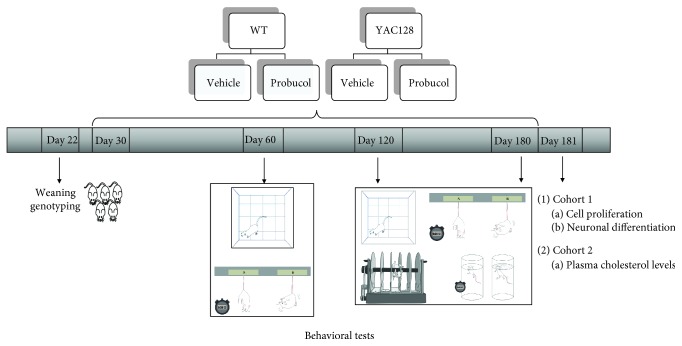
Experimental protocol. Thirty-day-old WT and YAC128 female and male mice received probucol (30 mg/kg/day) or vehicle (1% CMC) in drinking water for 5 months. Two distinct cohorts of animals were used. Animals from Cohort 1 were submitted to the tail suspension test (TST) and open field test (OFT) at 2, 4, and 6 months of age and to the forced swimming test (FST) and the rotarod test at 4 and 6 months. 24 h after the last behavioral test, mice were transcardially perfused; their brains were removed and processed for immunohistochemistry analyses of cell proliferation and neuronal differentiation. Animals from Cohort 2 were subjected to the same treatment regime and euthanized 24 hours after the last behavioral test to determine cholesterol plasma levels.

Probucol (Sigma, St. Louis, MO, USA; 30 mg/kg/day) was diluted in carboxymethylcellulose (CMC), 1 mg/mL. The probucol dose was chosen based on previous studies that reported neuroprotective effects of this compound [48–50]. YAC128 and wild-type (WT) mice were randomly divided into four experimental groups (with equal numbers of males and females included in each group, 10 mice/group): 1 (WT vehicle), 2 (YAC128 vehicle), 3 (WT probucol), and 4 (YAC128 probucol). Probucol-treated WT and YAC128 mice received probucol in drinking water for 5 months (from 1 to 6 months of age). Vehicle-treated WT and YAC128 mice received vehicle (CMC) in drinking water during the same period of time (Figure 1).

### 2.3. Behavioral Analyses

YAC128 and WT mice treated with either vehicle or probucol were submitted to the open field test followed by the tail suspension test (TST) at 2, 4, and 6 months of age. The forced swim test (FST) and the rotarod test were also performed at both 4 and 6 months (Figure 1). Mice tested at 2, 4, and 6 months of age were on vehicle or probucol treatment for 1, 3, and 5 months, respectively.

#### 2.3.1. Open Field Test (OFT)

To assess the effects of probucol on exploratory capacity, mice were evaluated in the OFT as previously described [51]. Mice were individually placed in a wooden box measuring 40 × 60 × 50 cm. The distance traveled and the time spent in the center of the arena were measured during a 6 min period. Tests were recorded using a digital video camera (HD Pro webcam C920 Logitech, CA, USA) and analyzed using the ANY-maze video-tracking system (Stoelting Co., Wood Dale, IL, USA).

#### 2.3.2. Rotarod Test

To evaluate the effects of probucol on the development of motor deficits, the rotarod test was used as previously described [52, 53]. The rotarod apparatus consisted of a rod 30 cm long and 3 cm in diameter that was subdivided into four compartments (Insight^®^, São Paulo, Brazil). Before the test, mice were allowed to train for 60 s, at a constant speed of 5 rpm, for acclimation to the equipment. After a 2 h resting interval, each mouse was tested for four 5 min sessions on the rotarod, with a gradual acceleration rate from 5 to 37 rpm during each session and with a 30 min resting period between sessions. The latency to the first fall from the rotarod and the number of falls during each 5 min session were recorded.

#### 2.3.3. Tail Suspension Test (TST)

The TST is useful in the screening of potential antidepressant drugs and other manipulations expected to affect depressive-like behaviors [54, 55]. In the TST, mice are placed in an inescapable and moderately stressful situation. In this test, immobility is interpreted as the absence of escape-related behaviors or helplessness. Briefly, acoustically and visually isolated mice were suspended 50 cm above the floor by adhesive tape placed approximately 1 cm from the tip of the tail for 6 minutes. Tests were recorded using a digital video camera (HD Pro webcam C920 Logitech, CA, USA) and analyzed by a highly trained observer blinded to the animals' identities. Animals were considered immobile when they hung passively and completely motionless. The total duration of immobility was measured according to the method described by [56].

#### 2.3.4. Forced Swimming Test (FST)

Mice were individually forced to swim in an open cylindrical container (diameter, 10 cm; height, 25 cm) filled with water maintained at 25°C. The total duration of immobility during a 6 min period was scored. Mice were judged to be immobile when they ceased struggling and remained floating motionless in the water, making only those movements necessary to keep their heads above the water [57].

### 2.4. Volumetric Analysis and Evaluation of Endogenous Hippocampal Cell Proliferation and Neuronal Differentiation

#### 2.4.1. Tissue Processing

Following behavioral analyses, one cohort of 6-month-old animals (*n* = 5 − 6 mice/group) was deeply anesthetized with an intraperitoneal (IP) injection of ketamine (100 mg/kg) and xylazine (8 mg/kg) and transcardially perfused with 0.9% sodium chloride followed by 4% paraformaldehyde (PFA). Brains were removed and left in 4% PFA overnight at 4°C and subsequently transferred to 30% sucrose. Following saturation in sucrose, serial coronal sections were obtained using a vibratome (Vibratome, Series 1000, St. Louis, MO, USA) at 30 *μ*m thickness. Sections were collected into a 1/6 section-sampling fraction and stored in azide solution (0.5%) at 4°C.

#### 2.4.2. Volumetric Analysis of the Total Hippocampus, the Dentate Gyrus Subregion, and the Striatum

Nissl-stained coronal sections were used to estimate the total volume of the total hippocampal formation, the hippocampal dentate gyrus (DG) subregion, and the striatum. Images were captured with a ZEISS Axio Scan.Z1 scanner (Jena, Thuringia, Germany). Using the software ZEN Wildfield 2012 Blue Edition, the area of the whole hippocampus, the DG subregion, and the striatum was measured in each coronal section using Cavalieri's principle. The volume was estimated using the following formula: ∑*A* × *T* × *I*, where ∑*A* is the sum of the areas of each region of interest (whole hippocampus, DG subregion, or striatum), *T* is the section thickness (30 *μ*m), and *I* is the number of section intervals (6).

#### 2.4.3. Immunohistochemistry

Two adjacent series of sections were processed for detection of the endogenous proliferative markers Ki-67, a nuclear protein that is expressed during all active phases of the cell cycle, but is absent from cells at rest [58, 59], and proliferating cell nuclear antigen (PCNA), which is expressed during all active phases of the cell cycle and for a short period of time after cells become postmitotic [60]. Briefly, brain sections were incubated in citric acid (dissolved in 0.1 m TBS, pH = 6.0) for 5 min at 95°C. This process was repeated twice to ensure complete unmasking of the nuclear antigens. After quenching with 3% H_2_O_2_/10% methanol in 0.1 m PBS for 15 min and preincubation with 5% normal goat serum (NGS) for 1 h, sections were incubated for 48 h at 4°C with either a rabbit polyclonal anti-Ki-67 primary antibody (1 : 500; Vector Laboratories, Burlingame, CA, USA) or a rabbit polyclonal antibody against PCNA (1 : 100; Santa Cruz Biotechnology, Santa Cruz, CA, USA). After rinsing, sections were then incubated for 2 h with the secondary antibody (biotin-conjugated goat anti-rabbit IgG, 1 : 200; Vector Laboratories). Sections were incubated with the avidin–biotin–peroxidase complex (Vectastain ABC Elite Kit PK4000; Vector Laboratories) for 1 h, and the bound antibodies were visualized using 2,2-diaminobenzedine (DAB; DAB kit SK 4100; Vector Laboratories) as the chromogen. Sections were mounted onto 2% gelatin-coated microscope slides and dehydrated through a series of ethanol solutions of increasing concentrations (50, 70, and 95%) followed by a 5 min incubation in xylene (Synth, Diadema, SP, Brazil). Finally, slides were coverslipped with Entellan mounting medium (Merck, Darmstadt, Germany).

An additional series of brain sections containing the hippocampus was processed for immunohistochemistry against doublecortin (DCX), a microtubule-associated protein specifically expressed by newly differentiated and migrating neuroblasts (i.e., immature neurons) [61]. Briefly, after quenching with 3% H_2_O_2_/10% methanol in 0.1 m PBS for 15 min and preincubation with 5% normal horse serum (NHS) for 1 h, sections were incubated for 48 h at 4°C with a goat polyclonal anti-DCX primary antibody (1 : 400; C-18, Santa Cruz Biotechnology). Sections were then incubated for 2 h with the secondary antibody (biotin-conjugated horse anti-goat IgG, 1 : 200; Vector Laboratories). Bound antibodies were visualized as described above.

#### 2.4.4. Morphological Quantification

All morphological analyses were performed on coded slides, with the experimenter blinded to the identity of the samples, using an Olympus IX83 microscope (Olympus, Hamburg, Germany) equipped with 10x, 20x, and 40x objectives (Olympus CellSens microscope imaging software (CellSens Dimension 1.2, Olympus)). A Peltier-cooled digital camera (Olympus DP73, Olympus) was used for image capturing. A modified stereological approach was used to estimate the total numbers of Ki-67-, PCNA-, and DCX-positive cells present along the entire subgranular zone (SGZ) of the hippocampal DG as previously described [33]. All sections along the entire dorsal/ventral axis of the hippocampus that contained the DG subregion (i.e., from 1.34 mm posterior to the bregma to 3.52 mm posterior to the bregma [62]) were used for the analysis, resulting in 10–12 DG-containing sections per brain. All positive cells present along the entire SGZ of each DG section and located within two to three cell diameters below the granule cell layer (GCL) were counted. The results were expressed as the total number of labeled cells in the DG subregion of the hippocampus by multiplying the average number of labeled cells/DG section by the total number of 30 *μ*m thick-sections containing the DG (estimated to be 73 sections in the mouse brain), and these values were expressed by DG volume. Images were processed with Adobe Photoshop 4.0 (Adobe Systems, Mountain View, CA, USA). Only contrast enhancements and color level adjustments were made; otherwise, images were not digitally manipulated.

### 2.5. Total Plasma Cholesterol Levels

A separate cohort of 6-month-old animals (*n* = 5 mice/group) was euthanized by rapid decapitation for blood sample collection. The total plasma cholesterol levels were determined by an enzymatic colorimetric method, using commercial kit reagents (Labtest Diagnostica®, Lagoa Santa, MG, Brazil), according to the manufacturer's instructions. The total cholesterol levels were expressed in mg/dL.

### 2.6. Statistical Analyses

All statistical comparisons were performed using the Statistica 10 analytical software (StatSoft Inc., Tulsa, OK, USA). Results were expressed as mean ± standard error of the mean (SEM). Behavioral data were analyzed with repeated measures analysis of variance (ANOVA). Histological (i.e., volumetric) and immunohistochemical data were analyzed with two-way ANOVA for genotype and treatment followed by the Duncan post hoc test when appropriate. A *P* value of < 0.05 was considered to be statistically significant.

## 3. Results

### 3.1. Effects of Chronic Probucol Treatment on Depressive-Like Behavior in YAC128 Mice

**Figure 2:**
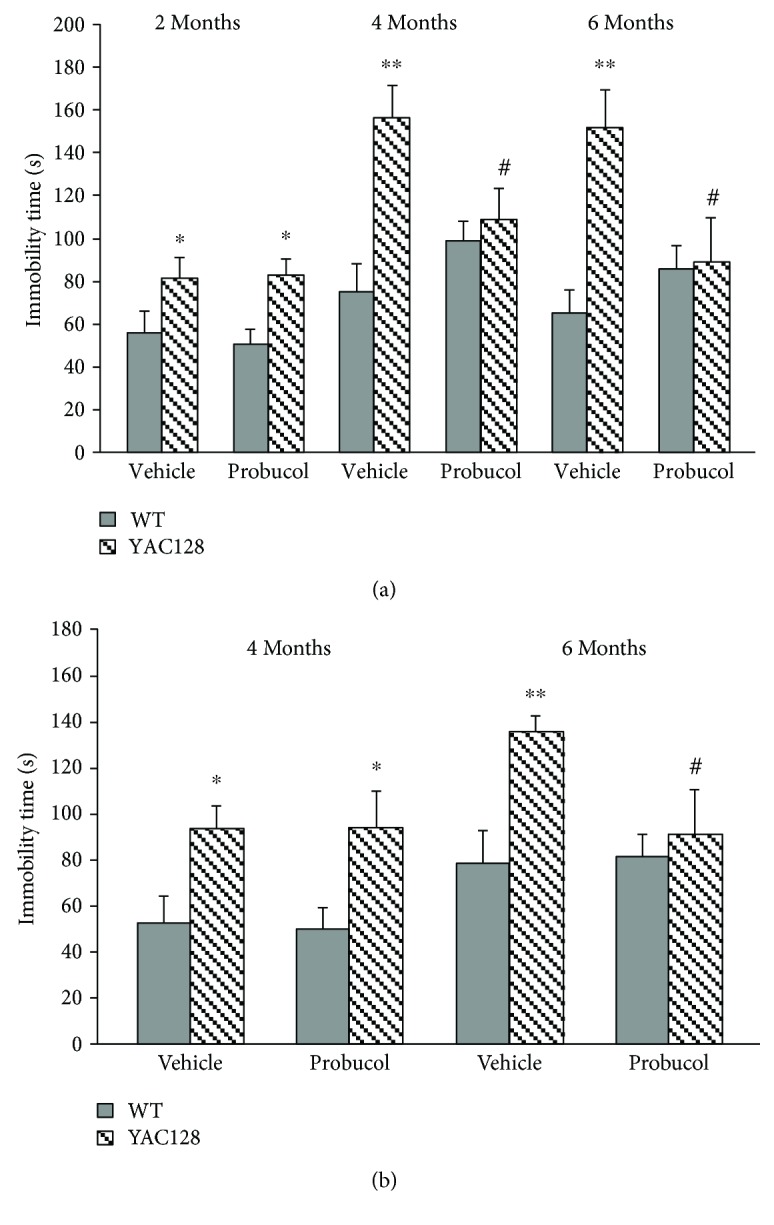
Antidepressant-like effects of chronic probucol treatment (30 mg/kg/day) on WT and YAC128 mice as assessed with the tail suspension test and forced swimming test. The total immobility time in the TST (a) and FST (b) was determined, and values are represented as mean ± SEM (*n* = 10 mice/group). Results were compared with repeated measures ANOVA followed by the Duncan post hoc test. ^∗^ *P* < 0.05 and ^∗∗^ *P* < 0.01 when compared to vehicle-treated WT animals, and ^#^ *P* < 0.05 when compared to vehicle-treated YAC128 mice.

To assess the occurrence of depressive-like behaviors, YAC128 and their age-matched WT counterparts were subjected to the TST at 2, 4, and 6 months of age and to the FST at 4 and 6 months of age. Repeated measures ANOVA indicated a significant main effect of genotype at 2, 4, and 6 months of age [*F*(3, 34) = 8.21, *P* < 0.01], with YAC128 mice showing an increase in immobility when compared to WT mice at all time points tested (2 months: *P* < 0.05 and 4 and 6 months: *P* < 0.01). In addition, a significant genotype versus probucol interaction was also noted [*F*(3, 34) = 4.31, *P* ≤ 0.01]. Further post hoc analyses indicated that treatment with probucol was able to reverse the depressive-like behavior exhibited by YAC128 at 4 (*P* ≤ 0.01) and 6 (*P* < 0.01) months of age, as demonstrated by a significant decrease in the immobility time in the TST (Figure 2(a)). With regard to the FST, repeated measures ANOVA indicated a significant main effect of genotype [*F*(2, 35) = 9.91, *P* < 0.01], with YAC128 mice showing an increase in immobility when compared to WT mice at both 4 and 6 months of age (4 months: *P* < 0.05 and 6 months: *P* < 0.01). Further post hoc analyses indicated that treatment with probucol was able to reverse the depressive-like behavior exhibited by YAC128 at 6 months of age (*P* < 0.05), as demonstrated by a significant decrease in the immobility time in the FST (Figure 2(b)).

### 3.2. Effects of Chronic Probucol Treatment on Motor Ability in YAC128 Mice

**Figure 3:**
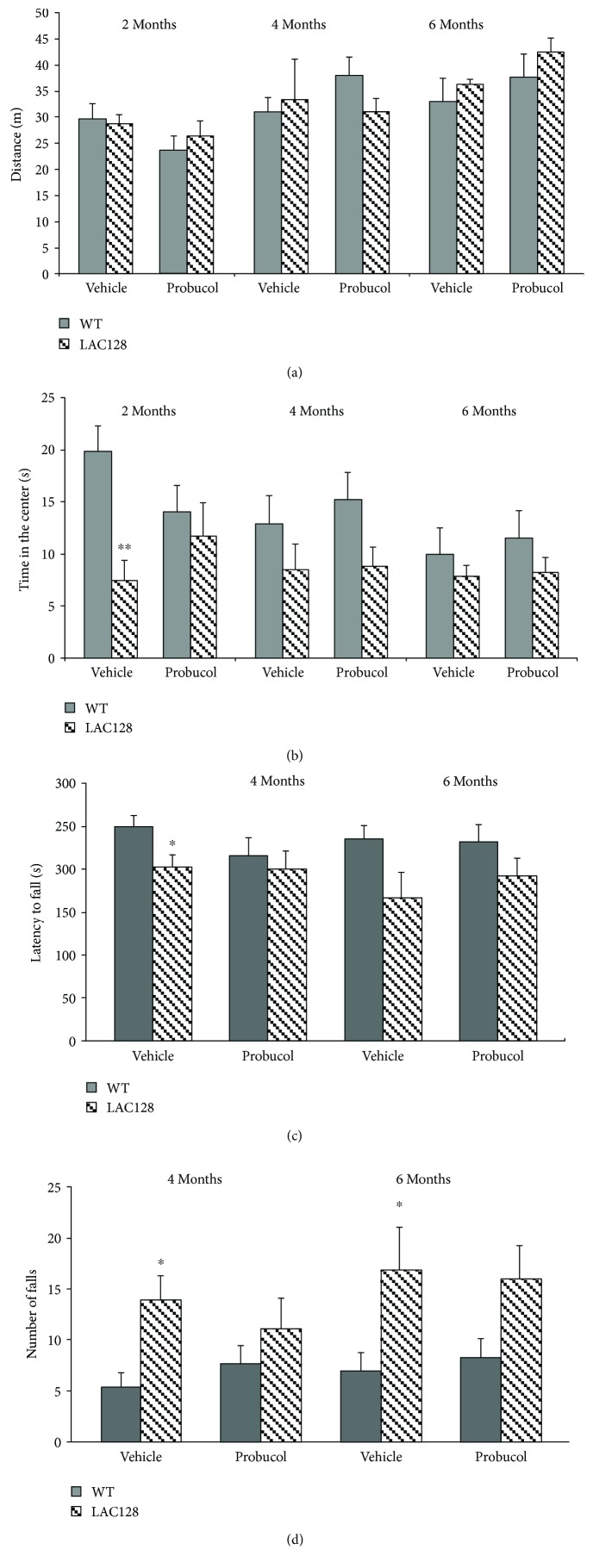
Effects of chronic treatment with probucol (30 mg/kg/day) on locomotion and motor balance in WT and YAC128 mice as assessed with the open field test (a and b) and the rotarod test (c and d). The distance traveled in the open field (a) and the time spent in the center of the open field (b) and the latency for the first fall (c) and the number of falls (d) in the rotarod were evaluated, and values are represented as mean ± SEM (*n* = 8–10 mice/group). Results were compared with repeated measures ANOVA followed by the Duncan post hoc test. ^∗^ *P* < 0.05 when compared with the WT group and ^∗∗^ *P* < 0.01.

To discard any potential effects of probucol treatment on exploratory capacity and overall activity (which could potentially affect performance in the TST and FST), the distance traveled (in meters) and the time spent in the center of the arena during a 6 min period were assessed by the OFT. A repeated measures ANOVA failed to detect any statistically significant effects of treatment [*F*(3, 34) = 1.72, *P* = 0.18] and genotype [*F*(3, 34) = 0.42, *P* = 0.73] and no significant genotype versus probucol interaction [*F*(3, 34) = 0.40, *P* = 0.75] on this parameter. Thus, YAC128 mice at 2, 4, and 6 months of age showed similar exploratory behavior and overall activity as their age-matched WT controls, regardless of probucol treatment (Figure 3(a)). On the other hand, a repeated measures ANOVA revealed a significant effect of genotype with regard to the time spent in the center of the arena [*F*(3, 34) = 4.06, *P* = 0.01], with 2-month-old mice spending significantly less time in the center when compared with their wild-type littermate controls (*P* < 0.01). However, no significant effect of treatment [*F*(3, 34) = 0.25, *P* = 0.86] and no significant genotype versus treatment interaction [*F*(3, 34) = 1.84, *P* = 0.16] were detected (Figure 3(b)).

To determine the effect of chronic probucol administration on motor coordination and balance, the performance of 4- and 6-month-old YAC128 and their age-matched WT controls was assessed in the rotarod. Repeated measures ANOVA revealed a significant main effect of genotype [*F*(2, 27) = 3.85, *P* < 0.05], but no significant main effect of treatment [*F*(2, 27) = 0.80, *P* = 0.45] nor a significant treatment versus genotype interaction [*F*(2, 27) = 0.50, *P* = 0.60] were detected with regard to the latency to the first fall (Figure 3(c)). Further post hoc analyses indicated a significant decrease in the latency to fall in 6-month-old YAC128 mice as compared with their WT littermates (*P* < 0.05). Similarly, a repeated measures ANOVA also revealed a significant main effect of genotype [*F*(2, 27) = 5.18, *P* ≤ 0.01], but no significant main effect of treatment [*F*(2, 27) = 0.01, *P* = 0.98] nor a significant treatment versus genotype interaction [*F*(2, 27) = 0.65, *P* = 0.52] were detected with regard to the number of falls. Further post hoc analysis revealed an increase in the number of falls in both 4- and 6-month-old YAC128 mice as compared with their WT littermates (*P* < 0.05; Figure 3(d)).

### 3.3. Effects of Chronic Probucol Treatment on Hippocampal and Striatal Volume

**Figure 4:**
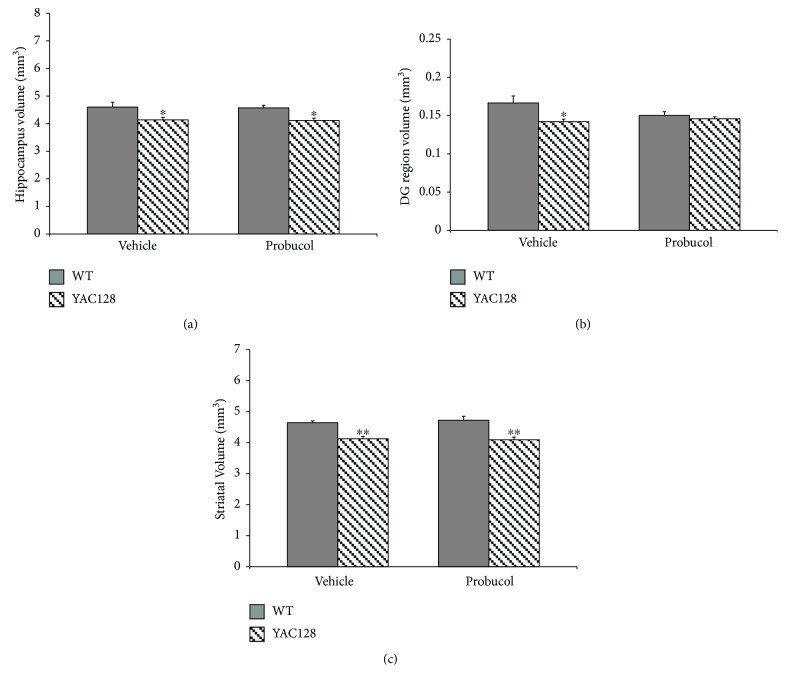
Volumetric analysis of the total hippocampus (a), the hippocampal DG subregion (b), and the striatum (c) in 6-month-old WT and YAC128 mice. Data are presented as mean ± SEM (4-5 mice/group), and results were analyzed with two-way ANOVA followed by the Duncan post hoc test. ^∗^ *P* < 0.05 and ^∗∗^ *P* < 0.01 when compared with the WT group.

To determine whether gross changes in the hippocampus and striatum could be detected during the early stages of disease progression in the YAC128 mouse model and whether probucol treatment could reverse such alterations, we estimated the volume of the whole hippocampus, its DG subregion (given its relevance for neurogenesis), and the striatum. A two-way ANOVA revealed a significant effect of genotype [*F*(1, 16) = 13.37, *P* < 0.01] but no significant effect of probucol treatment [*F*(1, 16) = 0.04, *P* = 0.83] nor an interaction between genotype and treatment [*F*(1, 16) = 0.001, *P* = 0.97] with regard to the volume of the whole hippocampus (Figure 4(a)). Further post hoc analyses indicated a significant decrease in hippocampal volume in 6-month-old YAC128 mice as compared with their WT littermates (*P* < 0.05). Similarly, a two-way ANOVA revealed a significant effect of genotype [*F*(1, 16) = 6.62, *P* < 0.05] but no significant effect of probucol treatment [*F*(1, 16) = 1.26, *P* = 0.27] nor an interaction between genotype and treatment [*F*(1, 16) = 3.27, *P* = 0.09] with regard to the volume of the hippocampal DG subregion (Figure 4(b)). Further post hoc analyses indicated a significant decrease in the volume of the DG subregion in 6-month-old YAC128 mice as compared with their WT littermates (*P* < 0.05). Moreover, a two-way ANOVA revealed a significant effect of genotype [*F*(1, 12) = 39.98, *P* < 0.01] but no significant effect of probucol treatment [*F*(1, 12) = 0.06, *P* = 0.81] nor an interaction between genotype and treatment [*F*(1, 12) = 0.35, *P* = 0.56] with regard to striatal volume (Figure 4(c)). Further post hoc analyses indicated a significant decrease in striatal volume in 6-month-old YAC128 mice as compared with their WT littermates (*P* < 0.01).

### 3.4. Effects of Chronic Probucol Treatment on Hippocampal Cell Proliferation on 6-Month-Old YAC128 Mice

**Figure 5:**
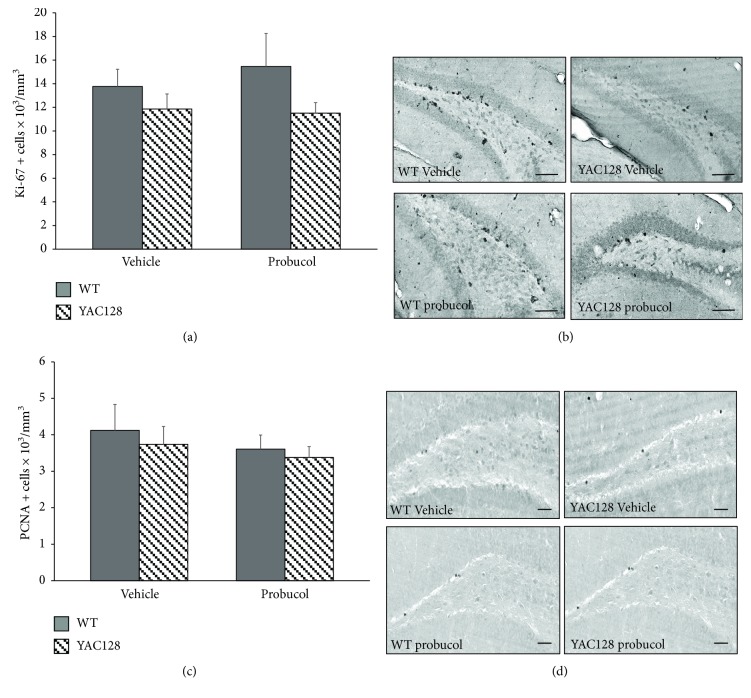
Evaluation of cell proliferation in the dentate gyrus of 6-month-old WT and YAC128 mice treated with probucol using endogenous cell cycle markers. Cell proliferation in the SGZ of the hippocampal DG was assessed by Ki-67- (a and b) and PCNA- (c and d) immunohistochemistry. Data are presented as mean ± SEM (*n* = 6 mice/group), and results were analyzed with two-way ANOVA followed by the Duncan post hoc test. Representative photomicrographs of Ki-67 (b) and PCNA (d) expression in the hippocampal DG of YAC128 and WT mice treated with either vehicle or probucol (scale bar = 50 *μ*m).

To analyze the potential effects of probucol on DG cell proliferation in YAC128 mice, we used the endogenous proliferation markers Ki-67 and PCNA [58–60]. A two-way ANOVA revealed no significant effects of genotype [*F*(1, 20) = 2.79, *P* < 0.11] and treatment [*F*(1, 20) = 0.14, *P* = 0.70] and no significant interaction between genotype and treatment [*F*(1, 20) = 0.34, *P* = 0.56] with regard to the density of Ki-67-positive cells present along the entire SGZ of the hippocampal DG (Figures 5(a) and 5(b)). Similarly, no significant effects of genotype [*F*(1, 20) = 0.37, *P* = 0.54] and treatment [*F*(1, 20) = 0.77, *P* = 0.39] and no significant interaction between genotype and treatment [*F*(1, 20) = 0.02, *P* = 0.87] were observed with regard to the density of PCNA-positive mitotic cells present with the hippocampal DG of 6-month-old YAC128 and WT mice (Figures 5(c) and 5(d)).

### 3.5. Effects of Chronic Probucol Treatment on Hippocampal Neuronal Differentiation in 6-Month-Old YAC128 Mice

**Figure 6:**
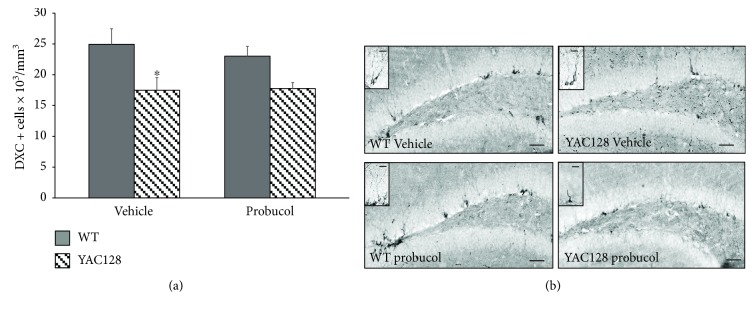
Evaluation of neuronal differentiation in the dentate gyrus of 6-month-old WT and YAC128 mice treated with vehicle or probucol using endogenous cell cycle markers. Neuronal differentiation in the SGZ of the hippocampal DG was assessed by DCX immunohistochemistry (a and b). Data are presented as mean ± SEM (5 mice/group), and results were analyzed with two-way ANOVA followed by the Duncan post hoc test. ^∗^ *P* < 0.05 when compared with the WT group. Representative photomicrographs of expression of DCX-positive cells (b) in the hippocampal DG of YAC128 and WT mice treated with either vehicle or probucol (scale bar = 50 *μ*m or 20 *μ*m, in detail).

To analyze the potential effects of probucol on DG neuronal differentiation in YAC128 mice, we used the endogenous marker DCX, a microtubule-binding protein that is expressed in newly differentiated and migrating neuroblasts [61]. A two-way ANOVA revealed a significant effect of genotype on the density of DCX-positive cells present along the entire SGZ of the hippocampal DG [*F*(1, 16) = 11.48, *P* < 0.01]. However, there was no significant effect of treatment [*F*(1, 16) = 0.18, *P* = 0.66] and no significant interaction between genotype and treatment [*F*(1, 16) = 0.33, *P* = 0.56] with regard to the density of DCX-positive neuroblasts. Further post hoc analyses revealed a significant decrease in the number of DCX-positive neuroblasts in 6-month-old YAC128 mice when compared to their WT littermates (*P* ≤ 0.01). However, probucol treatment during 5 months did not significantly affect the decline in DCX-positive cells observed in the YAC128 DG (Figure 6).

### 3.6. Effects of Chronic Treatment with Probucol on Total Plasma Cholesterol Levels in YAC128 Mice

Plasma cholesterol levels in both WT and YAC128 (treated with either probucol or vehicle) are shown in Supplementary Figure 1. Two-way ANOVA revealed no significant effects of genotype [*F*(1, 16) = 1.88, *P* = 0.18] and treatment [*F*(1, 16) = 0.64, *P* = 0.43] and no significant genotype versus treatment interaction [*F*(1, 16) = 0.30, *P* = 0.58] with regard to plasma cholesterol levels.

## 4. Discussion

In the present study, YAC128 HD mice exhibited a depressive-like behavior as early as 2 months of age and this phenotype was maintained at least until animals reached 6 months of age, observed as a significant increase in the immobility time in the TST (at 2, 4, and 6 months of age) and FST (at 4 and 6 months of age). Previous studies have also observed the occurrence of depressive-like behaviors in this transgenic HD model starting at 3 months of age and progressing into later stages of the disease [18, 20, 22], and a recent study of our group has recapitulated this depressive phenotype in 3-month-old YAC128 mice [21]. In addition, several studies have reported the presence of depressive-like behaviors during the early stages of disease progression in other HD transgenic mouse models such as the R6/1, N171-82Q, Hdh^Q111/Q11^, and the bacterial artificial chromosome (BACH) models [22, 63–71]. Thus, the results reported in the present study corroborate these studies and further demonstrate that depressive-like behavior can be observed during the initial phase of the disease progression and as early as 2 months of age in the YAC128 HD transgenic mouse model. Of note, these results are in line with the symptoms observed in HD patients, highlighting the relevance of this transgenic mouse model in elucidating mechanisms concerned to the human disease.

The potential neuroprotective effect of chronic probucol treatment on mitigating the occurrence of depressive-like behaviors in YAC128 mice was also investigated. Both WT and YAC128 mice received probucol in drinking water between 1 (i.e., before overt behavioral symptoms began) and 6 months of age. Our results show that chronic treatment (3 and 5 months, resp.) with probucol was able to prevent the occurrence of depressive-like behaviors in this HD transgenic mouse model at both 4 and 6 months of age. This is the first study showing a positive effect of probucol on regulating affective behaviors in a transgenic HD mouse model. The reduction in the immobility time elicited by probucol cannot be attributable to a psychostimulant action of this compound. This conclusion derives from the fact that in our study, probucol treatment produced a significant decrease in the immobility time in the FST or TST and did not alter the locomotor activity in animals as compared to control animals. However, a possible limitation of our study is the fact that repeating the open field test in the same animals can affect the performance in the test. For example, a significantly decreased time in the central area was only observed in the YAC128 mice at two months of age but not at later time points when animals are reexposed to the open field arena.

In this study, YAC128 mice presented motor deficits at 4 and 6 months of age, as shown by a significant reduction in rotarod latency time as well as a significant increase in the number of falls. Previous studies have demonstrated the presence of motor deficits in this HD transgenic mouse model at 3, 4 [18–20], and 6 [16] months of age. Pouladi et al. assessed motor dysfunction in these animals at 3 and 12 months of age and observed a significant worsening of motor performance at more advanced ages, demonstrating the progressive nature of this motor deficit [20]. Studies with other HD transgenic mouse models have demonstrated the presence of motor deficits in the BACH mice at 4 months of age [18], in the R6/1 at 2–5 months of age [25, 72], and as early as 1 month in R6/2 mice [27]. Although probucol demonstrated a significant antidepressant-like effect, this compound was unable to reverse the motor deficits exhibited by YAC128 HD mice at both 4 and 6 months of age. Even though the reasons for this lack of effect are not currently understood, this confirms that distinct neuronal pathways and mechanisms mediate the depressive-like behaviors and motor deficits observed in YAC128 HD mice. Indeed, motor deficits are mainly a consequence of neuronal dysfunction in the striatum whereas the affective behaviors are likely the result of limbic system (i.e., hippocampal) dysfunction, and it is possible that probucol might act mainly by preventing hippocampal dysfunction (see discussion below). Nevertheless, probucol has been shown to attenuate motor impairment in a 3-nitropropionic (3-NP) lesion model of HD [47] and decrease hyperlocomotion induced by 6-hydroxydopamine (6-OHDA) in a mouse model of PD [46], showing that this compound might also be effective in improving motor deficits in models associated with more overt cell dysfunction and death (such as that observed in the 3-NP HD and 6-OHDA PD toxin-induced lesion models, which are characterized by a more acute damage).

Of note, the beneficial effects of probucol (a lipid-lowering compound) [73] were not associated with hypocholesterolemic effects, as no significant differences in the plasma levels of cholesterol were observed following 5 months of probucol treatment. Similar to various other studies [47, 74, 75], we did not find a significant effect of probucol treatment with regard to plasma cholesterol levels. There is a vast literature showing that the hypocholesterolemic effects of probucol are greatly dependent on the dose used, as well as on the animal species and/or strain employed. In fact, although probucol has been reported to display hypocholesterolemic effects in both humans and different animal species [73, 76], a significant number of studies were not able to replicate such effect. For example, [75] was unable to detect a significant effect of probucol treatment on serum cholesterol levels in rabbits following a 4-month treatment regimen (500 mg/kg/day). In line with this, in a key review article on the long-term use of probucol as a hypocholesterolemic drug, [77] reported that the results from different experimental and clinical studies with probucol were at times confusing and suggested that such discrepancies may be due, at least partly, to different experimental conditions, including type of subjects being studied (different animal species and/or strains and presence of diverse health conditions in humans), different diets, and varying treatment periods and drug doses. With respect to clinical studies, these same authors highlighted the fact that the hypocholesterolemic action of probucol is not equally observed in every patient [77].

The cardinal neuropathological characteristic of HD is striatal atrophy, with selective loss of striatal medium-sized gamma-aminobutiric acid (GABA)-ergic spiny neurons [78] with striatal neuronal atrophy and loss being strongly correlated with the development of the classic HD motor symptoms. Here, we found a significant decrease in striatal volume in 6-month-old YAC128 mice, a result that is in agreement with previous studies showing striatal atrophy in the YAC128 HD mouse model [16, 79–82]. However, as discussed earlier, other brain regions such as the hippocampus are also affected in HD [83–85], suggesting a role for this structure in affective disturbances, which are seen both in HD patients and transgenic mouse models for this disease. Indeed, we have also observed a decrease in the volume of the total hippocampus as well as of its DG subregion in 6-month-old YAC128 mice. Our results are in accordance with studies in diverse HD transgenic mouse models that have documented reduced cortical, globus pallidus, and hippocampal volumes [80, 86–89]. Moreover, in humans, Rosas et al. observed atrophy in several brain regions, including the hippocampus [85].

Furthermore, the hippocampus is one of the few regions of the brain that retains the ability to generate new neurons during adulthood. Several studies suggest that adult hippocampal neurogenesis plays a key role in psychiatric and neurological disorders, such as depression [90, 91]. Indeed, neuroimaging and meta-analysis studies have consistently demonstrated a reduction in hippocampal volume in individuals with depression [92–96]. In rodents, depression has been correlated with reduced adult hippocampal neurogenesis [97]. In addition, the fact that antidepressant treatment attenuates symptoms of depression while also positively regulating hippocampal neurogenesis [34, 35, 98] suggests a strong link between a reduction in adult hippocampal neurogenesis and the occurrence of depressive-like behaviors [99].

Given that YAC128 mice have decreased hippocampal cell proliferation and neuronal differentiation [21, 33] and that a deficit in neurogenesis has been postulated as an underlying cause of depression [97], in the present study, the effects of probucol treatment on adult hippocampal cell proliferation and neuronal differentiation in the YAC128 transgenic mouse model were also assessed. However, in the present study, no deficits in hippocampal cell proliferation (assessed with the Ki-67 and PCNA endogenous cell cycle markers) were observed in YAC128 mice at 6 months of age. This result is inconsistent with the work of Simpson et al., who observed a significant (albeit small) reduction in the number of Ki-67 positive cells but not of PCNA-positive cells in the hippocampal DG of YAC128 mice [33]. Other studies using truncated transgenic HD mice (R6/1 or R6/2) with ages between 2 and 5 months have also reported impairments in hippocampal cell proliferation using the endogenous cell cycle markers Ki-67 and PCNA [28], as well as the exogenous marker 5-bromo-2′-deoxyuridine (BrdU) [23, 26–28]. These discrepancies are likely due to differences between the transgenic mouse model used (the R6/2 model has a much faster and more severe disease progression [100], whereas the YAC128 model better replicates the slower disease progression observed in human HD patients [16, 38]) and how results are expressed in different studies (total cell numbers as in the case of the study by Simpson et al. [33] or cell densities as in the present study).

In addition, a significant decrease in the number of neuroblasts (DCX-positive immature neurons) in the YAC128 hippocampal DG was observed at 6 months of age. Again, these results are similar to those reported by Simpson et al., showing a significant decrease in hippocampal neuronal differentiation in this HD transgenic mouse model [33]. Other studies using truncated transgenic (R6/1 or R6/2) HD mice with ages between 1-2 months have also reported impairment in neuronal differentiation using the endogenous DCX marker [24, 28, 30, 31]. In contrast, Orvoen et al. failed to detect significant differences in the number of DCX-positive cells in the hippocampal DG of the truncated Hdh^Q111/Q111^ mouse model. Nevertheless, these authors observed a deficit in dendritogenesis in the hippocampus of Hdh^Q111/Q111^ mice [63], further supporting a compromised neuronal differentiation process in the HD brain. However, [64] observed no changes in neuronal differentiation (as assessed with DCX immunohistochemistry) in 3-month-old BACHD mice. Again, differences among the various transgenic mouse models and the genetic constructs they express are likely to account for these discrepancies. Moreover, we cannot exclude the fact that sex differences and/or estrous phase effects may have impacted the results reported here.

Probucol treatment for 5 months was unable to stimulate cell proliferation and hippocampal neuronal differentiation in 6-month-old YAC128 HD mice. Although probucol had no effect during these stages of the neurogenic process on the YAC128 mouse model, multiple mechanisms of action have been described for this compound, and therefore, it is likely that the beneficial effects reported in this study were mediated by an alternative mechanism. Indeed, probucol is a molecule with well-established anti-inflammatory and antioxidant properties [43–45, 47, 101–105], which is able to modulate the activity of endogenous antioxidant enzymes [44, 45, 47, 106], promote synaptic plasticity [42, 44], and increase the levels of brain-derived neurotrophic factor (BDNF) [43]. Of note, the antioxidant effect of probucol has been documented both in humans [102] and in animal models [44, 46]. Indeed, both *in vitro* and *in vivo* studies have demonstrated beneficial effects of probucol on models of neurodegenerative diseases such as AD [42, 44, 45], PD [46], and HD [47, 105], as well as cerebral endothelial dysfunction [43] and brain ischemia [48].

The etiology of neuronal loss in HD has not been fully elucidated, and therefore, several mechanisms have been proposed to contribute to neuronal dysfunction and death in the HD brain, including oxidative stress, synaptic dysfunction, and neurotransmitter dysregulation (e.g., glutamate-mediated excitotoxicity and dopamine-mediated toxicity), as well as a decrease in trophic support (namely, a reduction in BDNF levels; for review, see [107]). Previous studies have suggested that oxidative stress might not play a major role during the early and mild stages of disease progression in the YAC128 HD mouse model [108], and therefore, it is unlikely that the beneficial effects of probucol observed on the present study are mediated by a decrease in oxidative stress. Nevertheless, it is worth mentioning that probucol was able to counteract motor impairments and oxidative stress in a 3-NP lesion model of HD [47] as well as a 6-OHDA-lesion model of PD [46]. Again, this might be related to the fact that toxin-induced lesion models (such as the 3-NP HD and the 6-OHDA PD models) are associated with more acute and overt neuronal dysfunction and death as well as a marked increase in oxidative stress.

Synaptic dysfunction has been demonstrated in several animal models of HD [84, 109, 110], including early to mildly symptomatic YAC128 mice [111], and therefore, it is possible that the beneficial effects of probucol were the result of improved hippocampal synaptic plasticity. Of note, Santos et al. reported a significant decrease in the levels of synaptophysin in the hippocampus of a mouse model of AD (A*β*1–40), a deficit that was reversed by probucol treatment [44]. In addition, this compound was also able to increase hippocampal levels of synaptosomal-associated protein 25 (SNAP-25) in 26-month-old rats [42]. SNAP-25 and synaptophysin are synaptic markers closely associated with synaptic vesicles and crucial for the processes of neurotransmission, synaptogenesis, and dendritic remodeling [112]. Whether such processes are impaired in the hippocampus of mildly symptomatic YAC128 mice is not currently known, and future studies are thus warranted to further elucidate this.

Stress, impaired neurogenesis, and defects in synaptic plasticity represent three interconnected factors that are associated with depression [113–115]. As a matter of fact, early-symptomatic YAC128 mice show alterations in short-term synaptic plasticity [111], and it is possible that probucol might have ameliorated these alterations, thus contributing to the antidepressant-like behavior observed. On the other hand, alterations in monoaminergic metabolism and neurotransmission (which are thought to contribute to the etiology of depression) have been extensively reported in human HD brains [116–120], and various preclinical studies have found a correlation between altered monoaminergic neurotransmission and the occurrence of depressive-like symptoms in transgenic mouse models of HD [67, 121]. Whether probucol can modulate monoaminergic neurotransmission is currently unknown, and future studies are warranted to test this hypothesis. Finally, although the hippocampus is a major brain region in depression, other regions such as the prefrontal cortex, the cingulate cortex, the striatum, the amygdala, and the thalamus [122] have also been implicated in this mood disorder. All these regions are highly interconnected through complex neuronal circuits, and mood disorders are thought to alter these circuits. Thus, it seems reasonable to speculate that depression associated with HD may independently affect several of these circuits. Indeed, it is possible that the antidepressant-like effect of probucol may be related, at least in part, to the structural and functional preservation of these neuronal networks.

## 5. Conclusion

The results presented here demonstrate, for the first time, the beneficial effects of chronic probucol treatment on the occurrence of depressive-like behaviors in the YAC128 transgenic mouse model of HD. These beneficial effects of probucol were not related to an increase in hippocampal progenitor cell proliferation and neuronal differentiation. While future studies will further elucidate the underlying neuroprotective mechanisms of this compound, the present study indicates that probucol may be an effective modulator of depressive-like behaviors commonly observed during the early stages of HD.

## Data Availability

Requests for access to the data generated during this study can be obtained by contacting the authors of the study.

## Supplementary Materials

Supplementary Materials Supplementary Figure 1: effects of chronic probucol treatment on cholesterol plasma levels in 6-month-old YAC128 mice and their WT littermate controls. Cholesterol levels are expressed as mg/dL. Values represent means ± SEM (*n* = 5 mice/group). No significant main effects of genotype and treatment and no significant interaction between genotype and treatment were found with regard to plasma cholesterol levels.
