# Supplementary figures and images for: Antidepressant Effects of Probucol on Early-Symptomatic YAC128 Transgenic Mice for Huntington's Disease
Source: Neural Plast. 2018 Aug 14;2018:4056383. doi: 10.1155/2018/4056383 (PMC6112232; doi:10.1155/2018/4056383)

## Slide 1
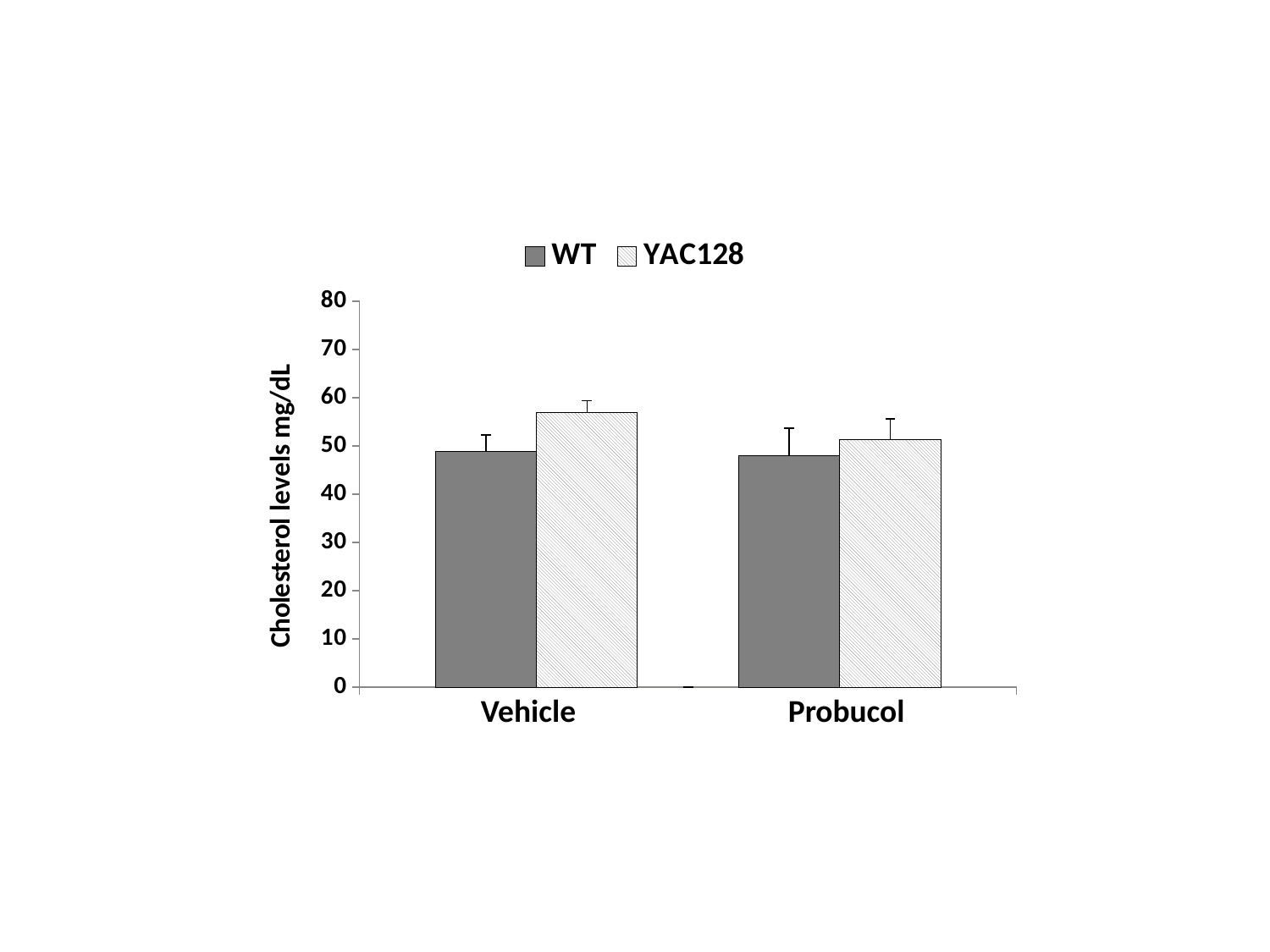

### Chart
| Category | | | | | |
|---|---|---|---|---|---|
Probucol
Vehicle

Supplement: Supplementary Materials — Supplementary Figure 1: effects of chronic probucol treatment on cholesterol plasma levels in 6-month-old YAC128 mice and their WT littermate controls. Cholesterol levels are expressed as mg/dL. Values represent means ± SEM (n = 5 mice/group). No significant main effects of genotype and treatment and no significant interaction between genotype and treatment were found with regard to plasma cholesterol levels. [file 4056383.f1.pptx]
